# Interactions between Autophagy and Bacterial Toxins: Targets for Therapy?

**DOI:** 10.3390/toxins7082918

**Published:** 2015-08-04

**Authors:** Jacques Mathieu

**Affiliations:** 1 Department of Microbiology, Institut de Recherche Biomédicale des Armées (IRBA), BP 73, Bretigny-sur-orge cedex F-91223, France; E-Mail: jacques.mathieu@irba.fr or jacques.mathieu@pasteur.fr; Tel.: +33-660-234-814; 2 Laboratoire Pathogénie des Toxi-infections Bactériennes, Institut Pasteur, 28 rue du Docteur Roux, Paris cedex 15 F-75725, France

**Keywords:** bacterial toxins, autophagy, cyclic AMP, therapeutic targets

## Abstract

Autophagy is a physiological process involved in defense mechanisms for clearing intracellular bacteria. The autophagic pathway is finely regulated and bacterial toxins interact with this process in a complex manner. Bacterial toxins also interact significantly with many biochemical processes. Evaluations of the effects of bacterial toxins, such as endotoxins, pore-forming toxins and adenylate cyclases, on autophagy could support the development of new strategies for counteracting bacterial pathogenicity. Treatment strategies could focus on drugs that enhance autophagic processes to improve the clearance of intracellular bacteria. However, further *in vivo* studies are required to decipher the upregulation of autophagy and potential side effects limiting such approaches. The capacity of autophagy activation strategies to improve the outcome of antibiotic treatment should be investigated in the future.

## 1. Introduction

**Figure 1:**
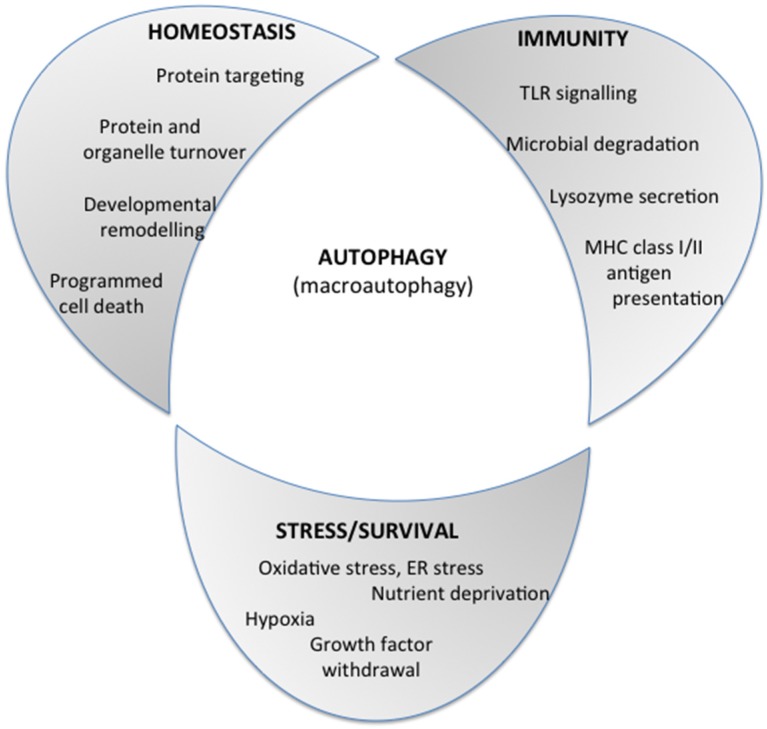
A schematic diagram, taken from [1], depicting the key roles of autophagy in cellular processes, such as homeostasis, responses to stress, the survival pathway, and immunity to bacterial pathogens. Reproduced from [1]. Copyright 2015, Elsevier.

Autophagy is a physiological process involved in regulating the overlapping or closely intertwined homeostasis, immunity and stress/survival responses (Figure 1). It also plays a major role in a broad range of human diseases, including cancer and neurodegenerative diseases [1,2].

Autophagy is a tightly regulated process [3,4,5,6]. It is involved in the survival of cells subjected to different stress conditions, such as starvation. If autophagy levels are excessively high, the cell may undergo autophagic cell death, also known as type II programmed cell death (PCD), a process different from apoptosis (type I PCD) and necrosis [7,8,9,10]. 

Autophagy consists of several different pathways: microautophagy, chaperone-mediated autophagy, lipophagy, macroautophagy and xenophagy. Microautophagy involves the direct uptake of cytosol, inclusions (e.g., glycogen) and organelles (e.g., ribosomes, peroxisomes) into the lysosome/vacuole by the protrusion, invagination or septation of the sequestering organelle membrane [11,12,13]. Mitophagy is a selective degradation of mitochondria mediated by micro- or macroautophagic processes [14]. Chaperone-mediated autophagy (CMA) is an autophagic process in mammalian cells by which proteins containing a particular pentapeptide motif related to KFERQ are transported across the lysosomal membrane and degraded [15,16,17]. The translocation process requires the action of integral lysosome-associated membrane protein type 2 (LAMP-2A) and the 73 kD cytosolic and lysosomal lumenal cognate heat shock proteins (hsc73) [18,19]. Lipophagy is a selective degradation of lipid droplets by lysosomes, contributing to lipolysis (breakdown of triglycerides into free fatty acids). In mammals, this selective degradation has been found to occur via macroautophagy (macrolipophagy) [20]. Macroautophagy is an autophagic process involving the formation of an autophagosome, a double- or multiple-membrane cytosolic vesicle of non-lysosomal/vacuolar origin [13]. Xenophagy is a cell-autonomous innate immunity defense mechanisms in which cells eliminate intracellular pathogens by capturing them in autophagosomes for subsequent killing [21]. In higher eukaryotes, the lysosome is a degradative organelle that compartmentalizes a range of hydrolytic enzymes and maintains a very low pH [13].

The elimination of intracellular pathogens by autophagy in mammalian cells (xenophagy) results not only in the degradation of invading bacteria, viruses, fungi and parasites, but also in the release of metabolites used by the pathogen during infection, thereby promoting cell survival. As recently reported by Devenish and Lai [22], the induction of autophagy results in the clearance of some bacterial pathogens, whereas other bacteria can manipulate autophagy for their own benefit and appear to replicate effectively within autophagosome-like vesicles. 

Three different autophagic processes can be distinguished in relation to bacterial infections of mammalian cells: macroautophagy (xenophagy) [23], non-canonical autophagy [24] and microtubule-associated protein 1 light chain (LC3)-associated phagocytosis (LAP) [25]. These processes will be described in detail below. 

Numerous bacterial pathogens interfere with the autophagy process, naturally involving defenses against intracellular bacteria. However, it has recently been shown that autophagy may play different roles after bacterial infection [26]: bacterial clearance, the coordination of cell-autonomous signaling, and in some cases, the promotion of bacterial replication.

The ability to survive within cells is crucial for several pathogenic bacteria. Following the invasion of their eukaryotic target cells, these bacteria are internalized within a membrane-bound vacuole that becomes more acidic as it develops into a mature degradative phagolysosome [27]. Some pathogens survive in this niche by either preventing vacuole-lysosome fusion or by modifying the environment within the phagolysosome. Certain bacteria have evolved means of escaping from the vacuole and continuing their life cycle within the cytosol [28,29,30]. Only a small number of bacteria are adapted for growth within the cytosol (reviewed in [31]).

Intracellular bacterial pathogens produce many virulence factors and toxins able to interfere with autophagy. These bacterial products include lipopolysaccharides (LPS), membrane-spanning secretion apparatuses such as those of *Salmonella* serovar *Typhimurium* and *Mycobacterium tuberculosis*, pore-forming toxins, such as those of *Streptococcus pyogenes* and *Listeria monocytogenes*, and bacterial adenylate cyclases. LPS is one of the most immunostimulatory components of the outer membrane of Gram-negative bacteria [32,33], capable of interfering with the autophagy machinery [34,35].

Many pathogenic bacteria produce pore-forming toxins (PFT), which are important virulence factors. These toxins are secreted as soluble monomers by the bacterium. They bind to a membrane receptor and multimerize, generating an amphipathic structure that serves as a pore. The cholesterol-dependent cytolysins, which use cholesterol as receptors [36], form a large family of pore-forming toxins produced by numerous bacteria, including *Bacillus*, *Staphylococcus*, *Clostridium*, *Streptococcus*, and *Listeria*. More than 600 putative bacterial adenylate cyclases have been identified [37], and cAMP seems to play a critical role in the autophagy pathway, as highlighted in a recent review [1].

This review focuses on two key questions: (1) Are the different interactions between autophagy and bacterial toxins involved in bacterial pathogenicity? (2) What therapeutic potential do molecules targeting the autophagy machinery have in cases of bacterial intoxication? 

A brief overview of the mechanisms involved in autophagic pathways is required, to clarify the potential interactions between bacterial toxins and autophagy. However, we do not provide an exhaustive description of these mechanisms here, as autophagic pathways are complex and not all the detail is necessary to understand possible interactions. Many molecular agents are involved and the number of publications in this field is growing.

## 2. Overview of Autophagy Mechanisms

Autophagy is a physiological process in which cytoplasmic components, including organelles and intracellular microbes, are engulfed by single- or double-membrane vesicles and targeted for destruction by fusion with a lysosome (review in [1,38]). Autophagy adjusts cellular biomass and function in response to diverse stimuli, including infection [39]. It plays specific roles in shaping immune system development, fueling host innate and adaptive immune responses, and directly controlling intracellular microbes through cell-autonomous innate defense. As an evolutionary counterpoint, intracellular pathogens have evolved to block autophagic microbicidal defense and to subvert host autophagic responses for their own survival or growth. The ability of eukaryotic pathogens to deploy their own autophagic machinery may also contribute to microbial pathogenesis. Thus, a complex interplay between autophagy and microbial adaptations to autophagy governs the net outcome of host-microbe encounters.

Pathogen engulfment is initiated by the formation of a phagophore or isolation membrane that engulfs the cytoplasmic components to form a sealed double-membrane structure called an autophagosome. This structure fuses with endosomes or multivesicular bodies to form the amphisome, which then fuses with the lysosome to generate the autolysosome, in which sequestered material is degraded by the lysosomal proteases. Certain microbes make use of lipid raft components to enter the host cell and trigger autophagy [40]. Over 30 components of the molecular machinery involved in autophagy have been identified [3], including a large number described as autophagy-related proteins (Atgs) [41]. The mammalian ortholog of the yeast Atg8 protein, microtubule-associated protein 1 light chain 3 (LC3), is an important element of this machinery and a key marker of autophagosomal compartments [42]. The LC3 family includes LC3A, LC3B and LC3C. These proteins are involved in the biogenesis of autophagosomes, and in cargo recruitment [43]. Vertebrate LC3 is regulated by phosphorylation of the *N*-terminal helical region by protein kinase A [44]. After translation, LC3 is proteolytically processed by Atg4, which cleaves a *C*-terminal glycine to generate the LC3-I form. Subsequently, when autophagy is activated, LC3-I binds covalently to the lipid phosphatidylethanolamine to generate the membrane-bound LC3-II form [42]. This processing is accomplished by a ubiquitin-like conjugation system consisting of Atg7 and Atg3 (E1-like and E2-like enzymes, respectively) and the Atg5-Atg12-Atg16L1 complex (E3-like enzyme) [45].

Three different autophagy processes are triggered by bacterial infections of mammalian cells. The first of these processes is macroautophagy (xenophagy), which involves the recognition of intracellular bacteria and their sequestration in double-membrane vesicles called autophagosomes. The cellular machinery responsible for autophagosome production has recently been comprehensively reviewed [23]. LC3 is a key component and signature marker of the autophagosomal membrane. This protein must be conjugated to phosphatidylethanolamine for incorporation into the isolation membrane. During isolation membrane expansion, the cargo (bacteria in this context) is sequestered and enclosed within the autophagosome. Autophagosomes subsequently fuse with lysosomes, to become autolysosomes, the contents of which are then degraded. The degradation products are then transported to the cytosol for reuse by the cell.

The second process is non-canonical autophagy [24,46,47,48]. This pathway has the same basic structure and function as macroautophagy, but not all the Atg proteins are required to form a functional autophagosome. Moreover, the direct recruitment of a set of Atg proteins to preexisting membranes has been described [49].

**Figure 2:**
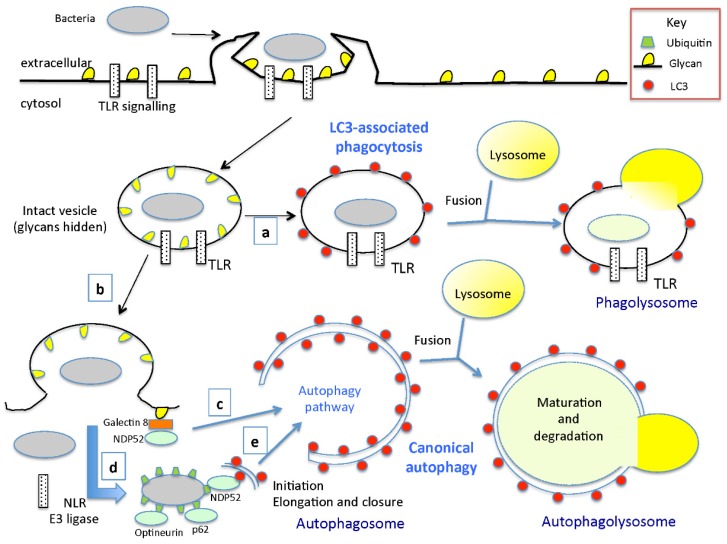
A simplified overview (a modified schema from [38,50,51] of LC3-associated phagocytosis (LAP) and canonical autophagy. (**a**) LAP is triggered by Toll-like receptors (TLRs) and other pattern recognition receptors (PRRs) in response to microorganisms, such as bacteria, that are taken up by phagocytosis or that have actively invaded non phagocytic cells. LAP requires a subset of autophagy genes for the labelling of phagosomes with LC3 (Atg8), which promotes their lysosomal delivery and the efficient killing of vesicular pathogens; (**b**) Damage of the limiting membrane of the pathogen-containing vesicle, either accidental or caused by pathogens attempting to escape from the vesicle, exposes the cytosol to glycans previously hidden inside the vesicle; (**c**) Cytosol-accessible glycans are detected by galectin-8 (danger receptor), which, by recruiting the cargo receptor NDP52 (nuclear dot protein 52), triggers autophagy; (**d**) Pathogens having escaped galectin-8-induced autophagy are met by another layer of PRRs in the cytosol such as NLR, and a yet-to-be-identified E3 ubiquitin ligase causes the ubiquitin-coating of invading bacteria. It remains to be established whether this ligase only targets membrane-associated or also free-floating bacteria, whether it is a PRR, and also whether its substrate is of bacterial [52] or host origin such as LRSAM1 [53] or WWP1 [54]; (**e**) A dominant pathway in the autophagic capture of bacteria such as *Salmonella* relies on tagging bacteria with a poly-ubiquitin coat, which is then bound by three apparently non-redundant ubiquitin binding autophagy adaptors, NDP52, p62 and optineurin [55,56,57,58]. As pointed out by Mostowy *et al.* [59], the recruitment of p62 and NDP52 to *Shigella* is interdependent. These adaptors subsequently recruit specific autophagic machinery components, such as the LC3/Atg8 family proteins, triggering the autophagic cascade and autophagosome formation (initiation, elongation and closure).

The third process is LC3-associated phagocytosis (LAP), which involves the direct recruitment of LC3 to single-membrane phagosomes [25]. LAP is initiated following infection with several bacterial pathogens, including *Escherichia coli* [60], *Salmonella serovar Typhimurium* [61], *Burkholderia pseudomallei* [62] and *Listeria monocytogenes* [63]. It requires some of the molecular machinery used for autophagosome formation, but the initiating ULK1 complex is dispensable [64]. In the context of bacterial infection, LAP is thought to ensure the rapid clearance of bacterial pathogens, by increasing phagocytosis and bacterial killing, as first demonstrated for *E. coli* [60]. Both autophagosomes and phagosomes undergoing LAP have LC3 in their membranes. One standard way of assessing autophagy involves determining the colocalization of bacteria with LC3, which is almost invariably detected as GFP-LC3 puncta. However, there is no readily applicable, simple technique for differentiating between the puncta corresponding to phagosomes and those corresponding to autophagosomes. The role of LAP in bacterial infections may therefore have been underestimated. Electron microscopy is the method of choice for distinguishing between single-membrane (phagosome) and double-membrane (autophagosome) compartments [65], but it is not always used by investigators. Figure 2 shows a simplified overview of LC3-associated phagocytosis (LAP) and canonical autophagy.

As pointed out by Lippai and Löw (2014) [66], the best known way to distinguish between the different cytoplasmic components destined for engulfment is to target properly labeled cargos to the inner surface of the growing phagophore. Precise delivery is generally ensured by interaction of the adaptor with both the membrane-anchored form of LC3, and the principal targets, which are mostly polyubiquitinylated. The recognition of ubiquitinylated proteins during autophagy is mediated by ubiquitin receptors interacting with ubiquitin-binding domains. p62, also known as SQSMT1, the first protein reported to have such an adaptor function [67], possesses a *C*-terminal ubiquitin-binding domain (UBA) [68] and a short LIR (LC3-interacting region) sequence responsible for LC3 interaction [67]. 

Ubiquitin-binding adaptor proteins, such as p62, NDP52 (nuclear dot protein 52) and optineurin, target many bacteria. p62 is required for the aggregation of ubiquitinylated proteins and plays a major role in their autophagic clearance [69,70]. Optineurin and NDP52 have recently been described as xenophagy receptors, making use of the autophagic machinery to eliminate ubiquitinylated intracellular pathogens [71]. The role of p62 in the regulation of autophagy remains a matter of debate. It has been suggested that it promotesTORC1 activation through involvement in its translocation to the lysosomal surface. Therefore, p62 reduction, like mTORC1 inactivation, may activate autophagy [72]. Moreover, p62 and NDP52 target intracytosolic *Shigella* to different autophagy pathways [59], and p62 also targets *Salmonella* [55,73]. One of the autophagy pathways to which *Shigella* is targeted by p62 and NDP52 is dependent on septin and actin [59]. Intracytosolic *Listeria* avoids recognition for autophagy by expressing ActA, a bacterial effector required for actin polymerization; p62 or NDP52 targets the *Listeria* ActA mutant to an autophagy pathway independent of septin and actin [59]. 

As highlighted by Sorbara and Girardin [74], it remains unclear how infected cells direct the phagophore to bacteria either free in the cytosol or confined to a vacuole without targeting other cellular constituents, such as organelles. This aspect is the subject of active investigations in the field of xenophagy. However, three other molecular mechanisms, in addition to ubiquitination, are known to confer bacterial targeting during xenophagy. These mechanisms involve: (i) Nod proteins; (ii) a galectin-dependent pathway and (iii) diacylglycerol (DAG). The Nod-like receptor (NLR) proteins Nod1 and Nod2 are cytosolic proteins that detect specific fragments derived from bacterial peptidoglycan and initiate pro-inflammatory signaling (NF-κB and MAP kinase pathways) [75]. Moreover, Nod1/2 interacts with the autophagy protein Atg16L1, driving recruitment of the autophagic machinery at the site of bacterial entry [76]. However, it is unclear whether Nod1/2 can detect bacteria, such as *Shigella* or *Listeria*, when they are free in the cytosol. Galectin proteins, such as galectin-8/3/9, are glycan-binding lectin proteins that are found in the cytosol and accumulate at sites of membrane damage due to *Salmonella* [77]. NDP52 is recruited to the *Salmonella*-containing vacuole (SCV) by galectin-8, through a mechanism different from the ubiquitin-mediated recruitment of NDP52 to the SCV [77]. Galectin proteins may be considered to be crucial sensors of the danger signals initiated at the damaged membrane by the exposure of glycans to the cytosol, these molecules normally being exposed to the vesicle lumen. 

The early accumulation of DAG around the SCV has been observed in *Salmonella*-infected cells [78] and the inhibition of DAG formation by pharmacological or genetic approaches results in the inhibition of bacterial autophagy [79]. The DAG-dependent and ubiquitin-dependent pathways of xenophagy, with targeting to the SCV, seem to be independent [79]. However, possible interdependence with other pathways remains to be assessed: see a review in [74]. It remains unclear whether DAG-dependent autophagy is a bacterial or host-initiated process [78].

One of the main features of autophagy is the dynamic rearrangement of cell membrane structures through multiple membrane fusion events. Soluble *N*-ethylmaleimide-sensitive factor attachment protein receptors (SNAREs) are known to play a key role in docking and the fusion of intracellular membranes. SNARE proteins include the vesicle-associated membrane proteins (VAMPs), which have glutamine as the central amino acid of the SNARE motif. VAMP3 and VAMP7 are thought to be involved in the fusion of autophagosomes and lysosomes. VAMP7 may be involved in phagosome formation during *Helicobacter pylori* infection, whereas VAMP3 is involved in phagosome maturation following *Mycobacterium tuberculosis* infection. *Yersinia pseudotuberculosis* has been shown to survive in murine bone marrow-derived macrophages (BMDMs), by hijacking autophagy and impairing autophagosome acidification: Atg5 knockdown facilitates *Yersinia* degradation [80]. This finding is consistent with the hypothesis that autophagy provides a replicative niche for this pathogen [80]. 

Numerous bacterial pathogens produce toxins that can interfere with host defense pathways. Interplay between autophagy and the regulation of pathogen replication is thought to occur [50]. Autophagy is an important cellular defense mechanism and a means of generating antigenic peptides for major histocompatibility complex (MHC) presentation. During bacterial infection, host cells can initiate autophagy to eliminate intracellular pathogens and/or toxins. The system may be more complex, involving the generation of inflammatory mediators, which may activate autophagy [81,82]. However, this process can also be exploited by microbes for survival and replication, potentially leading to host cell death [39].

The question of the role of bacterial toxins in triggering and regulating autophagy is considered in more detail below.

## 3. Are the Different Interactions of Autophagy and Bacterial Toxins Involved in Bacterial Pathogenesis?

There is evidence to suggest that the autophagy pathway plays a key role in innate immune defenses against intracellular pathogens (autophagy against intracellular pathogens is also called xenophagy). Antibacterial autophagy involves the selective recognition of intracellular bacteria and their targeting of the autophagy machinery for degradation [83].

### 3.1. Lipopolysaccharides 

Lipopolysaccharides (LPS) are among the most immunostimulatory components of the outer membrane of Gram-negative bacteria. LPS signals through the Toll-like receptor 4 (TLR4), inducing potent inflammatory responses and septic shock [84]. Autophagy has been shown to be involved in the secretion of proinflammatory cytokines in mice, through deletion of the gene encoding the autophagy protein Atg16L1, which triggers the production of large amounts of interleukin 1β (IL-1β) and IL-18 in response to LPS and other pathogen-associated molecular patterns (PAMPs) [85]. Atg16L1 has been identified as an Atg5-binding protein required for autophagy principally to regulate the location of the Atg12-Atg5 conjugate and for LC3 conjugation to phosphatidylethanolamine. Moreover, the depletion of autophagy proteins, such as LC3 and beclin-1, enhances the ATP-activation of caspase-1 and the secretion of IL-1β and IL-18 in a model of LPS-primed macrophages, and increases susceptibility to LPS-induced septic shock *in vivo*, in an experimental model of sepsis [86]. 

The autophagic machinery can be activated upon PAMP detection, by the cognate PRRs (pattern recognition receptors) [87]. This is an important barrier, particularly as there have been reports of an inability to detect macroautophagy downstream from TLR stimulation [85]. PRR signaling in autophagy may involve the complex reportedly formed between TLR adaptors (MyD88 and TRIF) and Beclin 1, and changes in the interaction of the antiapoptotic protein Bcl-2 with Beclin 1 upon TLR stimulation [88], akin to the Bcl-2-Beclin 1 interactions observed during the activation of autophagy by non-immunological signals [89].

Autophagy can also be regulated by reactive oxygen species (ROS) [90]. For example, the ROS produced by NADPH oxidase downstream from stimulation of the TLR or the Fcγ receptor in phagocytes has been shown to activate autophagy [61]. As previously described, LC3-II appears on phagosomes (LAP) without the formation of conventional double membranes, shortly after particle uptake, and the costimulation of TLRs by particles was responsible for initially drawing attention to the unconventional roles of Atg proteins [60]. However, the coactivation of NADPH oxidase and ROS production during the phagocytosis of opsonized or PAMP-laden particles may, in essence, mirror the observed induction of autophagy by ROS produced by mitochondria in response to starvation stimuli [90]. These events may best be understood within the concept of APMA (autophagic macrophage activation), a set of linked events in macrophages including connections between ROS production and autophagy. One recent review highlighted the crucial role of autophagy in inflammation and apoptosis in diseases [91]. The key role of mTOR in cellular metabolism is well known and its emerging role in antibacterial immunity is complex [92]. Several reports have linked immunity and metabolism [93,94]. The relationship between TLR signaling and mTOR activity was established in studies investigating the stimulation of innate immune cells with LPS. Rapamycin, a well-known inhibitor of mTOR activity, inhibits LPS-induced macrophage activation, and targets of mTOR signaling have been shown to be stimulated by LPS [95,96]. Two mTOR complexes have been identified: mTOR complex 1 (mTORC1), containing the Raptor protein and serving as the main mediator of metabolic homeostasis, and mTORC2 containing the Rictor protein and shown to act as the kinase complex for AKT [97]. Subsequent studies provided more insight into the mechanisms involved [98,99], with mTOR signaling described as regulated principally by TLRs, through two major pathways: an NF-κB-dependent pathway and a PI3K/AKT-dependent pathway. In the NF-κB pathway, the IKKβ protein, which is activated downstream from TLR signaling [100], may contribute to mTOR activation through the phosphorylation of tuberous sclerosis complex (TSC) 1, resulting in its inactivation. One recent study [101] described the reduction, by IKKβ inhibitors, of the LPS-induced phosphorylation of S6K1, a downstream target of mTOR, in MCF-7 cells. The second major link between TLR and mTOR signaling involves the PI3K/AKT pathway. LPS has been shown to induce TSC2 phosphorylation, which inactivates the TSC1/TSC2 heterodimer, leading to mTOR activation [102], and AKT inhibitors, which blunt TSC2 phosphorylation [103]. There is genetic evidence to suggest that the PI3K pathway acts as a critical negative regulator of the pro-inflammatory response, but the outcome of PI3K activation downstream from immune receptors depends on the type of cell activated (reviewed in [100]). As these data were obtained for different cell types, *in vivo* experiments are required to confirm the involvement of these pathways. 

Nod-like receptors constitute another type of PAMP detector: Nod1 detects moieties containing d-glutamyl-meso-diaminopimelic acid from Gram-negative bacteria [104], and Nod2 detects muramyl dipeptides from the peptidoglycans of certain bacteria [105]. The role of Nod-like receptors in mTOR signaling has received much less attention than that of TLRs (reviewed in [92,106]). However, some of the available data suggest that the intracellular sensors Nod1 and Nod2 are critical for the autophagic response to invasive bacteria. Nod1 and Nod2 recruit the autophagy protein ATG16L1 to the plasma membrane at the site of bacterial entry [76].

The outcome of LPS stimulation depends on the cell type studied and the orchestration and fine tuning of an ongoing immune response, with pleiotropic roles for the various molecular actors involved. Intracellular pathogens have developed virulence factors strongly involved in pathogenicity, such as toxins, to help them to survive after entering eukaryotic cells. 

### 3.2. Bacterial Pore-Forming Toxins (PFTs)

Pore-forming toxins (PFTs) are the most common class of bacterial protein toxins, and are important bacterial virulence factors [107]. They are secreted by the pathogens in a water-soluble form that binds to the target cell, generally leading to multimerization to form an amphipathic structure that inserts itself into the target cell membrane to form a pore. PFTs are classified according to the type of structure they use to insert into the lipid bilayer upon pore formation: α-PFTs cross the membrane as α-helices, whereas β-PFTs do so as β-sheets. The pore-forming colicins secreted by *Escherichia coli* are representative members of the α-PFT family [108]. A number of similar proteins that do not fully qualify as PFTs should also be mentioned. The most prominent of these proteins include the translocation domains of certain non-pore-forming toxins. In these so-called “AB” toxins, the B subunit is responsible for binding to the target cell and translocation of the A subunit into the cytoplasm. The A subunit has enzymatic activity. Examples include the diphtheria toxin, the translocation domain of which resembles colicins, and anthrax toxin, the B subunit of which forms a heptameric transmembrane channel similar to aerolysin, and the staphylococcal α-toxin channel. The B subunits of AB toxins do not insert into the membrane of target cells because they require an acidic environment. Anthrax toxin, secreted by *Bacillus anthracis*, consists of three protein exotoxins called the edema factor (EF), the lethal factor (LF) and the protective antigen (PA). Binary combinations that include EF+PA, referred to as the edema toxin (ET) and LF+PA, referred to as the lethal toxin (LT), are toxic ([109]. As described by Parker *et al.* [110], the two moieties required for toxicity are named A and B, by analogy to other toxins in which the A moiety bears the toxic enzymatic activity and the B moiety binds to the surface of the target cell and delivers the A moiety into the cytosol PA mediates translocation of the two alternative A moieties, LF and EF, into the cytosol. EF is an adenylate cyclase, which will be described in the next section. LF is a zinc metalloprotease, the canonical substrates of which are mitogen-activated protein kinase kinases, which it inactivates [111], thereby disrupting signaling pathways in host cells. As reported by Agarwal and Bishai, 2009 [112], LF can induce autophagy in mammalian cells, presumably to enhance the clearance of bacteria from the cytoplasm by diverting toxins to the autophagosome, where they are degraded, after lysosomal fusion. 

Cytolysin toxin (VCC), a pore-forming toxin from *Vibrio cholerae* [113], causes extensive vacuolation in epithelial cells. The relationship between the vacuolation caused by VCC and the autophagic pathway was investigated and the authors showed that treating cells with VCC increased the punctate distribution of LC3, which was colocalized with VCC-induced vacuoles, demonstrating the interaction of large vacuoles with autophagic vesicles. Electron microscopy confirmed that the vacuoles induced by VCC had the hallmarks of autophagosomes. Interestingly, the inhibition of autophagy leads to lower cell survival in the presence of VCC. VCC also fails to induce vacuolization in Atg5^−/−^ cells, and these cells have strongly impaired survival responses to this toxin. Thus, autophagy acts as a cellular defense pathway against secreted bacterial toxins, but it remains unclear whether these toxins have a dynamic relationship with autophagy. 

*Staphylococcus aureus* secretes a number of pore-forming toxins, such as α-toxin, which oligomerizes on binding to a target cell. The resulting oligomers then insert into lipid bilayers to form pores [110]. Pore-forming toxins induce a large decrease in intracellular ATP levels [114,115,116,117,118]. As reported by Kloft and colleagues [119], PFTs, such as *S. aureus* α-toxin, *V. cholerae* cytolysin (VCC), streptolysin O and *E. coli* hemolysin, induce the phosphorylation of AMPK in epithelial cells. Morphological features, such as multivesicular bodies and vesicles delineated by double membranes, have provided evidence for the induction of autophagy by PFTs [120]. Studies of several bacterial species, such as *Streptococcus pyogenes*, *Listeria monocytogenes*, *Shigella flexneri*, *Salmobella enterica* and *S. aureus* [121,122,123,124], have shown that selective autophagy can be induced by membrane damage and/or PFTs. Selective autophagy has been shown to correlate with the ability of intracellular *L. monocytogenes* and *S. aureus* to produce a PFT, listeriolysin O (LLO) and α-toxin, respectively [119,123,125]. As described earlier in another model of autophagy (a nitrogen starvation-induced autophagy in yeast) [126], plasma membrane perforation triggers a starvation response and autophagy involving the elF2 α-kinase GCN2 [119,120]. GCN2 (general control, non-derepressible 2) also called elF2α-kinase 4, acts as a nutrient sensor. The phosphorylation of elF2 α-kinase causes a general blockade of translation, but some genes are exempt from this process and are overexpressed. This so-called “integrated stress response” is thought to prevent the accumulation of unfolded proteins during stress, to conserve energy and to reprogram gene expression [119].

As recently reported [127], the cellular receptor A disintegrin and metalloprotease 10 (ADAM10) has been identified as the α-toxin receptor. α-toxin targets both endothelial cells and epithelial cells, upregulating the enzymatic activity of ADAM10 and culminating in disruption of the tissue barrier *in vitro* and *in vivo* [128,129]. Maurer and colleagues (2015) found that stronger ADAM10 expression in autophagy-deficient mice exacerbates *S. aureus* infection [130]. This finding confirms the vital nature of the interaction of the toxin-ADAM10 complex in bacterial pathogenesis. Moreover, Atg16L1 deletion in endothelial cells increases lethality *in vivo* and this effect in dependent on α-toxin [131].

Several authors investigating the interaction of bacterial toxins and autophagic process have shown that bacterial toxins provide support for bacterial replication. It has been known for some time [132] that *Listeria monocytogenes*, a Gram-positive bacterium, survives within cells by producing listeriolysin (LLO), a pore-forming cytotoxin allowing it to escape from phagosomes. 

*Listeria*, probably with low levels of LLO expression [37], is targeted by the LAP pathway via diacylglycerol enrichment and downstream reactive oxygen species production, to establish spacious *Listeria*-containing phagosomes (SLAPs), which serve as intracellular niches for the bacterium [63]. LLO is known to uncouple the pH gradients of the primary phagosome by creating small pores in the phagosomal membrane [133]. As reported by Birmingham *et al.* [134], LLO, which is sufficient and necessary for SLAP formation, may uncouple pH gradients accross SLAP membranes. These authors hypothesized that autophagy might be involved in SLAP formation, through the targeting of damaged phagosomes to prevent bacterial escape into the cytosol. Differences in LLO activity seemed to result in different fates for *L. monocytogenes* within host cells [134]. At high LLO concentrations, the bacteria escape rapidly from the phagosomes and *L.* *monocytogenes* grows rapidly in the cytosol. At low LLO concentrations, phagosomes might mature into SLAPs, leading to slow bacterial growth in vacuoles. According to this hypothesis [134], the host cell preserves its viability by preventing bacterial colonization of the cytosol, but is unable to eradicate the pathogen. Lafont’s group [135] recently published a paper on LC3-associated pathways. They reported that the phagocytosis of TLRs in murine macrophages triggered LC3 recruitment to phagosomes: *Listeria monocytogenes* expressing low levels of LLO is not prone to phagosome escape and resides in single-membrane, LC-3 positive, spacious *Listeria*-containing phagosomes. Gupta *et al.* [136] showed that the clearance of *Listeria monocytogenes* from macrophages required interferon regulatory factor 8 (IRF8)-dependent activation of autophagy genes, followed by the autophagic capture and degradation of *Listeria* antigens.

VacA, a key toxin in *H. pylori* pathogenesis [137] that induces the formation of large vacuoles in intoxicated cells, is secreted as a monomer via a type IV autotransporter. It then inserts into the host cell membrane and assembles into dodecamer structures. VacA oligomers form chloride-selective membrane channels, which are essential for many of the cytotoxic effects in intoxicated cells. The 88-kD monomer can be cleaved into two subunits, p33 and p55. Structural data suggest that p33 forms the central pore, with p55 protruding outward into the lipid bilayer.

VacA can impair autophagic flux [138]. Conversely, autophagy can promote VacA degradation at early stages of infection, as shown by the enhanced accumulation of VacA in Atg5-deficient mouse embryonic fibroblasts (MEFs) [138]. Early VacA degradation by autophagy may limit the ability of VacA to access the cellular compartments/cofactors required to inhibit autophagic flux. 

*Serratia marcescens*, a Gram-negative bacterium that is an opportunistic pathogen in humans, produces numerous exotoxins, including the Sh1A pore-forming toxin. One very recent study [139] has shown that this toxin can elicit an autophagic response in host epithelial cells, highlighting its functional importance in the life cycle of *Serratia*. However, the link between autophagy and pathogenicity has not been clearly demonstrated and further *in vivo* experiments are required to confirm these observations.

Yu *et al.* [140] showed that autophagy facilitates *Salmonella* replication in the cytosol of HeLa cells. *Salmonella enterica* serovar *Typhimurium* is a facultative intracellular pathogen that contains two type III secretion systems (T3SSs) encoded by *Salmonella* pathogenicity islands (SPI-1 and -2). These T3SSs are necessary for *Salmonella* pathogenicity, as they deliver bacterial proteins to the host cell cytosol for the manipulation of various host cell pathways. The T3SS apparatus proteins InvA and SipB (a pore-forming toxin) and the effector SopB are required for the association of *Salmonella* with autophagosomes for replication in HeLa cells. These results challenge previous reports that p62 and autophagy protect host cells against *Salmonella* infection [55,124,141]. Through its pore-forming activity, SipB may damage *Salmonella*-containing vacuoles (SCV), allowing *Salmonella* to enter the host cell cytosol, where it can obtain access to the autophagy machinery. Further studies are required to account for these discrepancies and to decipher the complexity of the different interactions between intracellular bacteria, bacterial toxins and autophagy pathways.

### 3.3. Bacterial Adenylate Cyclases 

Many bacterial pathogens have evolved mechanisms for exploiting the regulatory functions of cAMP to suppress immune responses [142]. For example, *Bacillus anthracis*, *Bordetella pertussis*, *Pseudomonas aeruginosa*, *Yersinia pestis* and *Mycobacterium tuberculosis* produce adenylate cyclase toxins that increase intracellular cAMP levels in host cells. *Staphylococcus aureus* can synthesize adenosine, to increase cAMP levels in host cells through the activation of G-protein coupled adenosine receptors [143]. Most immunosuppressive phenotypes associated with toxins that increase cAMP levels have been attributed to the actions of either EPAC or PKA. The authors of this study showed that bacterial toxins that increase cAMP levels inhibit several types of autophagy, including antibacterial autophagy, in host cells. Two different bacterial toxins inducing increases in cAMP levels have been studied in particular detail: (i) edema toxin (ET) from *Bacillus anthracis*, formed from the edema factor (EF) and the protective antigen (PA). PA is required for the delivery of EF to the mammalian cytosol. EF is a calmodulin-dependent adenylate cyclase toxin (AC) that directly increases intracellular cAMP levels; (ii) cholera toxin (CT) from *Vibrio cholerae*, consisting of a component that functions as an ADP ribosyltransferase capable of increasing intracellular cAMP levels in an indirect manner. By contrast, both *B. anthracis* and *V. cholerae* express a second toxin, including the metalloprotease lethal factor (LF) and a PFT cytolysin (VCC) respectively, shown to modulate autophagy in target cells [113]. Moreover, the toxin factors secreted by bacteria, such EF and LF from *Bacillus anthracis*, can cooperate to suppress innate immune responses [144]. LC3 processing does not seem to be affected, suggesting that the toxins that increase cAMP levels disrupt a downstream step in autophagosome formation.

Table 1 shows examples of different factors potentially involved in the inhibition of autophagy initiation signaling, such as edema factor toxin from *B. anthracis* and cholera toxin from *Vibrio cholerae* [145] (lethal factors from *B. anthracis* and cytolysin from *V. cholerae* are not mentioned) and an unknown factor from *S. enterica* serovar *Typhimurium* that may affect the mTOR complex [146]. Some bacteria interfere directly with the activity of components of the autophagy pathways, for example, VirA from *Shigella flexneri*, which inhibits RAB1 in the host cell [147]. Moreover, some bacterial factors, such as IcsB from *S. flexneri* and ActA/lnlK (an internalin-like protein) from *L. monocytogenes*, enable the bacteria to evade autophagy recognition by masking the bacterial surface. IcsB has been shown to block bacterial engulfment by autophagosomes by camouflaging the bacterial surface protein VirG and inhibiting its interaction with Atg5 [148]. *Listeria monocytogenes* and *Shigella flexneri* evade autophagy by escaping from the vacuole by using factors (LLO, PI-PLC and IpaB) facilitating vacuole disruption [31,149,150,151]. Some bacteria escape autophagy by as yet undetermined mechanisms. For example, *Burkholderia pseudomallei* escapes autophagy by using various effectors [62,152] and *Francisella tularensis* does so using DipA [153,154].

**Table 1:** Examples of factors involved in the interplay between bacteria and autophagy, according to Huang & Brumell [155] and Asrat *et al.* [156].

Bacterium	Bacterial factors	Host factors	References
**Inhibiting autophagy initiation signaling**			
*Bacillus anthracis*	Edema factor toxin	cAMP	[145]
*Vibrio cholerae*	Cholera toxin	cAMP	[145]
*Salmonella enterica* serovar *Typhimurium*	Unknown	mTOR, RAG GTPases	[146]
**Directly interfering with the activity of autophagy components**			
*Shigella flexneri*	VirA	RAB1	[147]
**Evading autophagy recognition by masking the bacterial surface**			
*Shigella flexneri*	IcsB	Atg5 and septins	[59]
*Listeria monocytogenes*	ActA and lnlK	MVP and host factors that bind ActA	[157,158]
**Evading autophagy by escaping from the vacuole**			
*Listeria monocytogenes*	LLO (cholesterol-dependent cytolysin)	Membrane pore formation	[149]
*Listeria monocytogenes*	PI-PLC (phosphatidylinositol-specific phospholipase)	Facilitate vacuole disruption for escape	[31,150]
*Shigella flexneri*	IpaB (membrane pore formation)	Host cell invasion, membrane disruption and escape from the SCV	[31,151]
**Escaping autophagy by unclear mechanisms**			
*Burkholderia pseudomallei*	T3SS3 effector BopA and translocator BipD, T3SS1 ATPase encodded by bpscN	Unknown	[62,152]
*Francisella tularensis*	DipA	Unknown	[153,154]

As previously described, other pathogens, such as *S. aureus*, can synthesize adenosine, which seems to be required for escape from phagocytic clearance [143]. Extracellular adenosine can increase intracellular cAMP levels by activating cell-surface adenosine receptors [159]. The inhibition of autophagy by an increase in cAMP levels after the internalization of *S. aureus* may be involved in the escape mechanism, but this remains to be demonstrated. As reported in recent studies on yeast [160,161,162], PKA-specific phosphorylation sites have been identified on two critical autophagy proteins: Atg1 and Atg13. The PKA-mediated phosphorylation of these factors impaired the localization of Atg1 and Atg13 to the phagophore assembly site, resulting in an autophagy defect. The possible phosphoregulation of mammalian homologs of Atg1 and Atg13 by PKA remains to be investigated, and further studies are required to determine how the cAMP/PKA pathway affects the regulation of autophagy and its consequences for immunity in mammals. One particularly interesting study [163] showed that ET and CT induced a strong increase in cAMP levels in the perinuclear region. The authors of this study highlighted the importance of compartmentalization in cAMP-dependent signaling in many cell types. By contrast, they also showed that *Bordetella pertussis* adenylate toxin and forskolin (a pharmacological reagent used to increase intracellular cAMP levels) triggered an increase in cAMP concentration just below the plasma membrane [163]. ET and CT induce massive, long-lasting cAMP synthesis and PKA hyperactivation, triggering CREB phosphorylation and negative feedback activity. 

Nevertheless, the published findings for this study suggest that the inhibition of autophagy by toxins increasing cAMP concentration is of immediate benefit to the pathogen, as it blocks xenophagy, with the internalized bacteria thus avoiding degradation in the lysosome. Autophagy is required for the generation of antimicrobial peptides in lysosomes [164,165]. However, the precise role of bacterially induced autophagy inhibition in virulence remains incompletely understood, although a microarray analysis of *Francisella tularensis*-infected macrophages revealed that several autophagy genes were downregulated [166]. Nevertheless, *in vivo* experiments are required to affirm this hypothesis.

### 3.4. Other Virulence Factors 

Microbial pathogens that successfully parasitize eukaryotic cells (*i.e*., intracellular pathogens) have evolved in the setting of selective pressures imposed by cellular autophagy as a pathway central to innate and adaptive immunity. It is therefore unsurprising that microbes have developed several strategies for avoiding autophagolysosomal degradation and/or decreasing the autophagy-dependent activation of host immune responses. Researchers are beginning to decipher these molecular strategies and their potential roles in microbial pathogenesis, although our understanding of these aspects remains rudimentary in most cases. 

What appears more likely, at least based on the limited research to date, is that microbial adaptations suppressing the induction of autophagy may target some of the more general (*i.e.*, not pathogen-specific) signaling pathways involved in the up- or downregulation of autophagy. 

Another strategy employed by intracellular bacteria to escape the undesirable fate of lysosomal destruction is avoidance of capture by the autophagosome. Bacteria residing in phagosomes or other vacuolar compartments may seek to avoid lysosomal maturation, but the avoidance of autophagic capture may be particularly important for intracellular bacterial pathogens that escape into the cytoplasm. The evasion of autophagic capture has been described for at least three different intracytoplasmic bacteria: *Shigella flexneri*, *Listeria monocytogenes*, and *Burkholderia pseudomallei*. The first example to be described was the classic case, that of the escape of *Shigella* from autophagy. This escape seems to involve a particularly intriguing mechanism [148]. *Shigella* possesses a surface protein, VirG, required for actin-based motility. This protein binds to the autophagy protein Atg5, thereby targeting *Shigella* to the autophagosome. However, the bacterial T3SS effector, IcsB, binds competitively to Atg5, thereby hiding its own target molecule, VirG, and preventing autophagic capture. Together with the suppression of autophagosomal fusion with lysosomes and/or the acidification of pathogen-containing compartments, the bacterial autophagosomal-like compartments may enable the bacteria to persist in a nonacidic compartment. The enhanced pathogenicity of *L. monocytogenes* in mice with a macrophage-specific deletion of *Atg5* [167] demonstrates that *Atg5* expression in phagocytic cells is essential for cellular immunity to intracellular pathogens. In addition to shielding bacteria from the endolysosomal pathway, it has also been suggested that the localization of bacteria to LC3-positive compartments may allow cytoplasmic bacteria to regain access to the endocytic compartment, to promote bacterial egress through exocytosis (*i.e*., during *Francisella* infection) [154]. Other strategies also exist. For example, a streptococcal cysteine protease, SpeB, plays a critical role in the avoidance of ubiquitylation and recognition by the host autophagy marker LC3 and the ubiquitin-LC3 adaptor proteins NDP52, p62 and NRB1 [168].

Bacterial toxins and some virulence factors seem to interact with the autophagy machinery to allow the expression of bacterial pathogenicity. Several proteins involved in autophagic mechanisms, such as Atg5 and Atg16L1, seem to play a major role, as their deletion enhances pathogenicity [131,148,167]. As reported by Mostowy & Cossart in 2012 [124], autophagy controls the fate of a number of intracellular bacteria, including *Listeria*, *Shigella*, *Francisella*, *Salmonella* and *Mycobacterium*. By contrast, other bacterial pathogens, such as *Legionella*, *Coxiella*, *Yersinia*, *Brucella and Staphylococcus*, benefit from autophagy pathways and may subvert the autophagy machinery to favor the infection process. These alternative outcomes highlight the molecular complexity underlying bacterial autophagy, and suggest possible difficulties in the therapeutic modulation of autophagy to resolve bacterial infection. 

However, here we will analyze pharmacological strategies for enhancing or restoring autophagy processes to limit bacterial pathogenicity and to improve bacterial clearance.

## 4. What Therapeutic Potential Does the Targeting of Molecules Involved in the Autophagy Machinery Have for the Resolution of Bacterial Infections?

Here, we will focus on FDA-approved drugs and nutritional supplements, such as antioxidants and vitamins, already used in humans and likely to increase autophagy, which could therefore potentially be used to limit toxin effects, to prevent increases in bacterial numbers and to facilitate bacterial clearance (the contribution of autophagy upregulation to the therapeutic effects of these drugs for their currently approved clinical indications is unknown). Moreover, non-pharmacological interventions, such as caloric restriction and regular exercise, induce autophagy and may improve overall health [169,170].

In this review, antibodies able to block bacterial toxins specifically are not discussed, despite theour department’s development of monoclonal antibodies against anthrax toxins [171,172]. This strategy is very interesting but it requires the development of specific antibodies against each toxin, which may be very challenging. The development of specific inhibitors of enzyme activity for a given toxin, such as LT, is another strategy, involving the high-throughput screening of potential inhibitors [173]. It could be interesting to target an enzyme used by numerous bacteria, such as a specific inhibitor of bacterial adenylate cyclase. Finally, the targeting of bacterial toxins involved in autophagy induction might be a more interesting therapeutic approach than targeting proteins involved in the regulation of autophagy, but it may be difficult to find molecules able to counteract different bacterial toxins or to develop a specific anti-toxin molecule, which would be very expensive. One key challenge is the identification of new specific inducers of autophagy with fewer unwanted side effects than for available drugs. For the identification of new autophagy-inducing molecules, chemical screens can be performed, based on measurements of the fluorescence of autophagososmes (GFP-LC3-positive puncta) by live-cell imaging methods [174], FACS-based measurements of total levels of LC3 as readouts of autophagy [175,176,177], high-throughput screening approaches for identifying new regulators [178] or biochemical methods for the analysis of LC3 and p62 [179]. Proteomic mapping methods, such as spatially restricted enzymatic tagging, in living cells may be useful for the identification of autophagy-specific regulatory steps [180]. For example, as suggested by Levine *et al.* [181] targeting the kinase involved in phosphorylation of the autophagy receptor optineurin could enhance autophagic substrate clearance, including the clearance of ubiquitin-coated bacteria [182].

A synergistic strategy using a combination of antibiotic therapy and molecules targeting inflammatory processes was investigated by Popov *et al.* [183], who showed that the outcome of antibiotic treatment in a murine anthrax model could be substantially improved by combined administration of the caspase-1/4 inhibitor YVAD and the A3R agonist Cl-IB-MECA (chloro-6-[[(3-iodophenyl)methyl]amino]-9H-purin-9-yl]-1-deoxy-*N*-methyl-beta-d-ribofuranuronamide). The overall effect of this treatment is the inhibition of interleukin-1β-induced inflammation and activation of adenosine type-3 receptors (A3Rs). Treatments combining these molecules with ciprofloxacin resulted in up to 90% synergistic protection. All the untreated mice died, and antibiotic treatment alone protected only 30% of the animals. The authors of this study suggested that A3R activation inhibited the toxin-induced increase in intracellular cAMP levels, but no link to autophagy was described; this strategy is based on synergy with antibiotic treatment. Recent papers have described pharmacological agents targeting autophagy [181,184,185]. We focus here on FDA-approved drugs and nutritional compounds able to modulate autophagy. However, these products generally have pleiotropic actions, making it difficult to determine the contribution of the induction of autophagy to their therapeutic effects in patients. Preclinical studies [181] have demonstrated that some autophagy-inducing agents fail to to induce their beneficial effects in host organisms lacking autophagy genes. Autophagy enhancement has been reported to be useful in preclinical models of diseases: mTOR inhibitors in neurodegenerative diseases [186], tyrosine kinase inhibitors in diabetic nephropathy [187] and neurodegenerative diseases [188,189,190], carbamazepine in α1-antitrypsin deficiency [191], trifluoperazine in *Salmonella* infection [192] and statins in *Mycobacterium tuberculosis* infection [193]. It is unknown whether autophagy upregulation contributes to the therapeutic effects of these agents for their currently approved clinical indications. For acute infectious diseases, the non-autophagy-inducing actions may be tolerable if they are mild or apparent at doses substantially higher than those required to enhance autophagy. As shown in Table 2, currently available autophagy-inducing drugs and nutritional compounds can be grouped into four classes [2]:

**Table 2:** Selected FDA-approved drugs, pharmacological agents and nutritional compounds that modulate autophagy.

Autophagy inducers	Mechanism of action	References
**FDA-approved-drugs**		
Rapamycin	Induces autophagy by inhibiting mTORC1	[194,195,196]
Metformin	Upregulates AMPK, which promotes autophagy by inducing ULK1 phosphorylation	[197,198]
Isoniazid	Activates autophagy flux, oxidative stress and upregulates AMPK	[199]
Vitamin D3	Upregulates cathelicidin	[200,201]
Vitamin C	Antioxidant	[202]
Vitamin E	Antioxidant	[203]
Lithium	Lowers inositol and Ins(1,4,5)P" levels	[204]
Sodium valproate	Lowers inositol and Ins(1,4,5)P" levels	[196,204]
Carbamazepine	Lowers inositol and Ins(1,4,5)P" levels	[196,204]
Verapamil	Lowers intracytosolic Ca^2+^ levels	[196]
Clonidine and rilmenidine	Lower cAMP levels	[196]
Anti-psychotic drugs	Inhibit autophagy	[205]
Statins	Lower membrane cholesterol levels, thereby preventing cholesterol-dependent pore-forming toxins from forming pores	[206]
**Pharmacological agents**		
17-hydroxy-jolkinolide B	Activates heme oxygenase-1 expression	[207]
L-NAME	Decreases nitric oxide formation to induce autophagy	[208]
**Nutritional compounds**		
Resveratrol	Activates sirtuin 1 (histone deacetylase)	[209,210,211]
Epicatechins	Inhibit LPS-induced HMGB1 upregulation by stimulating its autophagic degradation	[212,213]
Catalase	Antioxidant	[214]
Chloroquine	Inhibits autophagosome-lysosome fusion	[215,216]
Vinblastine	Inhibits microtubule formation	[217]
Nocodazole	Inhibits microtubule formation and inhibits autophagosome-lysosome fusion	[218,219]
3-methyladenine, Wortmannin and LY294002	Inhibit phosphatidylinositol 3-3-kinase	[220]

AMPK: AMP-activated protein kinase; cAMP cyclic AMP; Ins(1,4,5)P_3_: inositol-1,4,5-triphosphate; L-NAME: *N*-l-arginine methyl ester; mTOR, mammalian target of rapamycin; mTORC1, mTOR complex 1; ULK1, Uncoordinated 51-like kinase 1; HMGB1, high mobility group B-1.

(1) Mammalian target of rapamycin complex 1 (mTORC1) inhibitors. Rapamycin (trade name Rapamune) and its analogs are macrolides produced by the bacterium *Streptomyces hygroscopicus*. Activators of AMPK, such as metformin (an antidiabetic drug) [221], and glucose starvation increase the AMP/ATP ratio, leading to an induction of AMP kinase activation, resulting in mTORC1 inhibition. Moreover, a diterpenoid from *Euphorbia fischeriana* that activates heme-oxygenase-1 (HO-1) expression [207] inhibits mTORC1 and enhances autophagy.

(2) Inhibitors of the phosphatidylinositol (PI) signaling pathway: lithium, valproic acid, carbamazepine. The PI signaling pathway is activated by cyclic AMP and EPAC (exchange protein activated by cAMP).

(3) Compounds linked in a potentially cyclic fashion via the pathway involving cyclic AMP, calcium and calpain: clonidine, rilmenidine and verapamil (agents used to treat hypertension and cardiac diseases), and anti-psychotic drugs.

(4) Other autophagy-modulating drugs: resveratrol, epicatechins, spermidine, vitamins, and inhibitors of nitric oxide formation, such as L-NAME.

### 4.1. mTOR Inhibitors

Mammalian target of rapamycin (mTOR), a serine-threonine kinase, is a central regulator of cellular metabolic homeostasis highly conserved throughout. The emerging role of mTOR signaling in the regulation of TLR-dependent innate responses and the activation of T cells and antigen-presenting cells has recently been reviewed [92], highlighting the importance of mTOR inhibition in the control of autophagy and intracellular bacterial clearance. As pointed out in previous publications [92], it is unclear whether bacterial pathogens can compete directly with infected host cells for nutrients, thereby contributing to the induction of metabolic stress pathways. It would be interesting to explore how drugs normally used to treat metabolic diseases influence the immune response. Metformin, for example, is used to modulate blood glucose levels and to inhibit mTORC1, which may induce inflammation. By contrast, it has been reported [222] that the treatment of *Shigella*-infected zebrafish with rapamycin may fail to promote host survival or bacterial clearance. This lack of efficacy of rapamycin may reflect effects on processes other than autophagy, because mTOR also affects many other cellular processes [223]. These findings indicate that caution is required concerning the therapeutic implications of autophagy upregulation. 

The relationship between autophagy and the inflammatory processes induced by bacteria is well documented [38], and autophagy has been reported to play a prominent role in pulmonary diseases [224]. The activation of autophagy by antibiotic (isoniazid) treatment has been shown to decrease the proinflammatory responses induced by *M. tuberculosis* (MTb) in macrophages [199]. A number of therapies increasing autophagy activity are effective against MTb infection. The antiprotozoan drug nitazoxanide, and its active metabolite tizoxanide, strongly stimulate autophagy, inhibiting mTORC1 signaling and the intracellular proliferation of MTb [225].

The recent discovery of a functional impairment of autophagy in cystic fibrosis [226] provides a new basis for understanding susceptibility to severe infections. Treatments that restore autophagy may improve pathogen clearance and decrease lung inflammation in the airways of CF patients.

By contrast, Aguirre and coworkers [227] showed that autophagy blockade enhanced the lung response to endotoxemia and starvation, a potent autophagic stimulus. This, in turn, decreased lung injury, as reported by another group [228].

Moreover, adaptive responses to sepsis [229] are required to prevent organ failure and death. Cellular signaling responses that limit cell death and structural damage allow cells to withstand sepsis and prevent irreversible organ dysfunction. One such protective pathway for reducing hepatocellular injury is the upregulation of HO-1 signaling. HO-1 is upregulated in the liver in response to multiple stressors, including sepsis and LPS, and has been shown to limit cell death. Pharmacological inhibition of HO-1 activity with tin protoporphyrin or by HO-1 knockdown prevents the induction of autophagic signaling in these models and results in greater hepatocellular injury, apoptosis, and death [230]. Furthermore, the inhibition of autophagy with 3-methyladenine or a small interfering RNA specific to VPS34, a class III phosphoinositide 3-kinase that acts as an upstream regulator of autophagy, results in hepatocyte apoptosis *in vivo* and *in vitro*. LPS induces the phosphorylation of p38 mitogen-activated protein kinase (p38 MAPK) at least partly by HO-1-dependent signaling. Moreover, the inhibition of p38 MAPK prevents CLP- or LPS-induced autophagy. Sepsis and LPS-induced autophagy protect against hepatocellular death, partly through HO-1 and p38 MAPK-dependent signaling. 17-hydroxy-jolkinolide B, a diterpenoid from *E. fischeriana*, triggers HO-1 production in LPS-stimulated macrophages, potentially leading to the inhibition of inflammatory mediators [207]. Further investigations are required to determine how autophagic signaling prevents apoptosis and cell death. 

### 4.2. Polyphenols

The engagement of TLRs or Fcγ receptors during phagocytosis induces the recruitment of the autophagy protein LC3 to phagosomes. TLR4 knockout mice have been reported to display lower levels of LT-induced cardiac dysfunction than control mice [231]. TLRs and Fcγ receptors are known to activate the NOX2 NADPH oxidase, which, through the release of ROS, play a major role in the killing of bacteria by phagocytes [61]. However, ROS overproduction can damage cells, as antioxidants can stimulate autophagy [232] and could be used as adjuvant treatments for infectious diseases [203,233]. Bacterial pathogens can manipulate autophagy by altering several processes, to ensure their survival and proliferation [234]. Autophagy may also act via an NADPH oxidase-dependent mechanism. 

Anthrax lethal toxin (LT), a critical virulence factor secreted by *Bacillus anthracis*, alters murine cardiomyocyte contractile function via an NADPH oxidase-dependent pathway [235]. LT has also been shown to induce the accumulation of reactive oxygen species (ROS) and autophagy in murine cardiomyocytes. In a model of cardiac catalase overproduction, catalase, an antioxidant enzyme, was found to attenuate LT-induced cardiac contractile and intracellular Ca^2+^ abnormalities. Catalase decreased the LT-induced increase in LC3-II levels, indicating a possible role for ROS in LT-induced autophagosome formation [214]. Moreover, Toll-like receptor 4 (TLR4) knockout was found to prevent LT-induced cardiac abnormalities, possibly through a mechanism associated with autophagy [231]. 

Kandadi’s group proposed an experimental murine model of cardiac-specific catalase overexpression protecting against *B. anthracis* LT, to improve antioxidant pathway targeting [214]. Another group of antioxidants, polyphenols from green tea, such as epigallocatechin-3-gallate (EGCG) was also tested. This antioxidant prevented death due to the effects of LT in rats [236]. This polyphenol is a well known anti-infectious compound [233,237]. However, some studies have shown that high concentrations of EGCG (100 µM) inhibit autophagy, leading to apoptosis in macrophage cell lines [238]. By contrast, low concentrations of EGCG (10 µM) induce autophagy, enhancing the degradation of LPS-induced aggregates of high mobility group B-1 (HMGB1), leading to anti-inflammatory effects [212]. As reported by Kim *et al.* [239], EGCG stimulates autophagy in endothelial cells via a Ca^2+^/CaMKKβ/AMPK-dependent mechanism (CaMKKβ = calmodulin-dependent protein kinase kinase β). Another polyphenol, resveratrol, induces autophagy in various models [232,240] and reduces endotoxin-induced cardiotoxicity [241]. In addition having antioxidant activity, resveratrol induces autophagy by activating a protein acetylase called sirtuin 1 [242,243,244]. It stimulates the deacetylation of cytoplasmic proteins by a sirtuin 1-dependent mechanism [243,245]. Pietrocola *et al.* [246] showed that a strong correlation and cause-effect relationship between cytoplasmic deacetylation reactions and the induction of autophagy could explain the effect of resveratrol. Only a few *in vivo* studies have been published and further studies of the utility of antioxidants for regulating the autophagic pathway are required. Moreover, a recent review [247] of the use of antioxidant therapy in cystic fibrosis reported poor evidence regarding clinical outcome. However, the mechanisms involved need to be determined and new pharmacological tools developed for testing in *in vivo* experimental models.

### 4.3. Vitamins

Another strategy is based on the stimulation of innate defense. Several authors [200,201] have reported that vitamin D3 induces autophagy in human monocytes/macrophages via cathelicidin, thus demonstrating a relationship relationship between vitD3-induced innate defense and autophagy activation. However, as highlighted in one study [248], limited data are available concerning the potential utility of pharmaceutical agents that induce autophagy as therapeutic agents for human infectious diseases. Other vitamins, such as vitamin C [202] and vitamin E [203], may have beneficial effects against sepsis, via the activation of autophagy and an increase in the elimination of damaged cell components.

**Figure 3:**
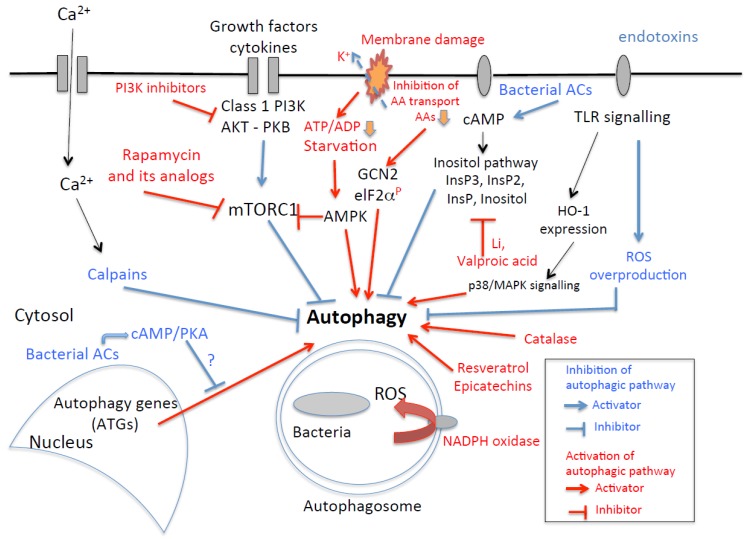
A simplified overview (a modified schema from [2]) of some molecular events (and potential drug targets) involved in bacterial autophagy. For example, the increase in intracellular calcium concentration leads to thea activation of calpains, which inhibit autophagy. Inhibitors of calcium or calpain could therefore interfere with the autophagic pathway. Inflammatory mediators, such as cytokines, trigger PI3K activation, leading to a decrease in autophage through effects on mTORC1. PI3K inhibitors or rapamycin treatment to inhibit mTORC1 enhances autophagy. Glucose starvation, which can be induced by stress, activates AMPK, leading to the inhibition of mTORC1 and the stimulation of autophagy. Membrane damage by pore-forming toxins induces starvation responses [120]. Loss of cellular potassium from perforated cells leads to the failure of nutrient transport and transient drop of ATP, thus activating cellular nutrient and energy sensors GCN2 and AMPK, subsequent phosphorylation of ElF2α and deactivation of mTORC1. Bacterial toxins, such as those with adenylate cyclase activity (ACs), enhance cAMP production, leading to an activation of the inositol pathway. InsP3 inhibits autophagy, and drugs such as lithium (Li) and valproic acid block the inositol cycle, restoring autophagy. The overproduction of cAMP activates the PKA pathway, potentially impairing the localization of Atg1 and Atg13 to the phagophore assembly site, resulting in defective autophagy. Motifs from bacteria or endotoxins are recognized by TLRs, which activate numerous pathways. For example, those triggering the HO-1 pathway can activate autophagy by activating mitochondrial ROS and p38 MAPK. However, endotoxins can induce oxygen stress and ROS overproduction, via the TLRs, either enhancing or inhibiting autophagy. Antioxidants, such as catalase and resveratrol, strongly enhance or restore the autophagic pathway.

Figure 3 shows a simplified overview of the regulation of bacterial autophagy (xenophagy) and potential drug targets likely to enhance autophagy. For example, numerous inhibitors of the cAMP/PKA pathway could be tested, to analyze their effect on the autophagic response and bacterial clearance. One recent study showed that a PKA inhibitor, H-89, could stimulate autophagy [249], but this effect appeared to be independent of PKA inhibition.

### 4.4. Adenosine Receptor Agonists

However, these pathways, involving numerous molecules, including mTORC1, can affect many processes other than autophagy. For example, they can inhibit AKT, a serine-threonine kinase involved in tumorigenesis and cell motility, thereby increasing susceptibility to cell death [250]. Data from Popov and coworkers [183] have shown that adenosine type-3 receptor agonists, such as *N*6-(3-iodobenzyl) adenosine-5'-*N*-methyluronamide can improve the outcome of antibiotic treatment in a murine model of anthrax. The authors put forward several possible explanations for their results, including AKT stimulation and cAMP downregulation. These agonists have been shown to protect against endotoxemia, and to decrease mortality in sepsis [251,252,253]. They have not been reported to affect autophagy, but the A1 adenosine receptor agonist (2 chloro-N6-cyclopentyladenosine) has been shown to stimulate the autophagic pathway [254]. As reported by Factor and coworkers [255], the A1R and A3R receptors inhibit adenylyl cyclase or lead to signaling through inositol-3-phosphate and phospholipase C in most cell systems. By contrast, the engagement of type 2 receptors activates adenylyl cyclase via Gsα and increases cAMP levels (for a review of the role of adenosine receptors in immune diseases, see [256]).

### 4.5. Antipsychotic Drugs

As reported by Shaw’s group [205], small-molecule inducers of basal autophagy have been characterized according to their effects on IL-1β production, autophagic engulfment and the killing of intracellular bacteria. These inducers include antipsychotic drugs, bromperidol, metergoline phenothiazine thioridazine, chlropromazine and clemastine (an antihistamine). Astemizole, a second-generation selective histamine H1-receptor antagonist that acts as a potent inducer of autophagy, significantly improves survival outcomes in a mouse model of prion disease [257]. The mechanism by which astemizole modulates autophagy has not been characterized, but this drug has been reported to have antifungal [258] and antimalaria [259] effects. Another small molecule, pimozide, an antipsychotic drug, has been reported to inhibit *L. monocytogenes* infection [260]. The inhibitory effect of pimozide on internalization was not specific to *L. monocytogenes*, as the phagocytosis of other bacterial species (*Bacillus subtilis*, *Salmonella entrica*, serovar *Typhimurium* and *E. coli* K12) was significantly inhibited in the presence of pimozide. Moreover, the antipsychotic drug thioridazine and the calcium channel blocker bepridil inhibit vacuolar escape and the intracellular replication of *L. monocytogenes* in a dose-dependent manner during the infection of murine macrophages [261]. Nevertheless, the surprising links between an antipsychotic drug, an antibacterial effect and the autophagic pathway require further exploration.

### 4.6. Statins

Cholesterol is a key component of autophagosome membranes, and drugs that modulate cholesterol turnover may play a major role in regulating autophagosome formation. Well known drugs, such as statins [193], act as competitive inhibitors of HMG-CoA reductase, a rate-limiting enzyme of the cholesterol biosynthesis pathway catalyzing the conversion of HMG-CoA reductase into mevalonate [262]. It has been suggested that statins have pleiotropic effects, including broad-range immunomodulatory and anti-inflammatory properties [263,264]. Statins have been shown to decrease mortality in patients with bacteremia [265] and to improve multiple organ dysfunction syndrome [266].

Simvastatin has been shown to increase protection against *Listeria monocytogenes* infection in mice, by counteracting *Listeria*-induced phagosomal escape [206]. Statins have been studied in diverse models of infection, including an experimental *Plasmodium berghei* cerebral malaria model in rodents. Statin treatment was found to improve the efficacy of antimalarial products, such as mefloquine, and to decrease neuroinflammation [267,268]. Further investigations are required to determine the role of autophagy in this model. 

The hypothesis that statin treatment can alter the effects of pore-forming toxins in host cells requires further investigation (the cholesterol-dependent cytolysin pore complex is reviewed in [269]), as these toxins contribute to the virulence of numerous bacterial pathogens [270]. 

According to Rubinsztein [2], caution is required when considering the use of autophagy-inducing agents for the treatment of patients with infections that may be alleviated by the upregulation of autophagy (such as tuberculosis). This is particularly true if these patients are coinfected with other pathogens that can exploit the autophagy pathway. Another potential concern is that excessively high levels of autophagy may exacerbate certain infectious diseases. For example, the inhibition of autophagy has been shown to improve acute lung injury caused by avian influenza A H5N1 infection [271]. The autophagic response in septic lungs is a protective response. However, autophagy following excessively high levels of autophagosome accumulation may be maladaptive in the late stage of sepsis, ultimately leading to acute lung injury [272].

Following on from studies of the autophagic pathway, a new generation of vaccines [31] could be developed for the future. These vaccines could include attenuated vaccines already in use and produced from bacterial strains specifically constructed so as not to replicate within the host cell or spread from cell to cell. Bacteria could be engineered to prevent their entry into the cytosol, through the introduction of genes mediating vacuolar lysis. Moreover, the delivery of antigens into this vacuolar compartment should enhance the presentation of class I major histocompatibility complex molecules and elicit cytotoxic T-cell responses.

As reported by Schein *et al.* [273], a new strategy for counteracting bacterial adenylate cyclase activity involves a combination of structural and computational approaches. This approach involves the selection of small organic molecules binding to residues within the substrate binding pocket of an adenylate cyclase, such as *B. anthracis* EF, from large, web-based compound libraries, on the basis of their docking scores. A family of fluorenone-based inhibitors has been identified, the members of which inhibit the release of cAMP from cells treated with EF. The lead inhibitor was also shown to prevent the diarrhea caused by enterotoxigenic *E. coli* (ETEC) in a murine model, possibly by serving as a quorum sensor. The ability of these inhibitors to inhibit anthrax could be tested in animal models, along with their ability to inhibit toxins similar to EF, such as toxins from *B. pertussis* or *V. cholerae*. 

Finally, cells or their products could be used to obtain an antibacterial effect. It has recently been reported [274] that mesenchymal stem cells (MSCs) can modulate autophagy in a parkinsonian model, and MSCs have also been reported to increase the antibacterial activity of granulocytes [275]. The mechanisms involved in the effects of MSCs on antibacterial activity, based on cell contact or soluble factors, need to be deciphered, and this will require determination of the precise role of autophagy in these processes. 

## 5. Conclusions

Autophagy represents a host defense pathway against pathogens and bacterial products, such as LPS, PFTs and bacterial adenylate cyclases. Autophagic pathways are altered by the bacterial toxins involved in pathogenicity, but other host cell signaling pathways also enhance bacterial survival within the cell. Further studies are required to decipher the mechanisms of pathogen recognition and host cell responses, for the development of new pharmacological strategies to use in conjunction with antibiotics against bacterial pathogens. 
